# Methotrexate Associated Renal Impairment Is Related to Delayed Elimination of High-Dose Methotrexate

**DOI:** 10.1155/2015/751703

**Published:** 2015-06-21

**Authors:** Shi-Long Yang, Fen-Ying Zhao, Hua Song, Di-Ying Shen, Xiao-Jun Xu

**Affiliations:** Division of Hematology-Oncology, Children's Hospital, Zhejiang University School of Medicine, Hangzhou 310003, China

## Abstract

Although Methotrexate (MTX) is an effective drug for the treatment of acute lymphoblastic leukemia (ALL), the toxicity remains a significant problem. In this prospective study, fifty-four patients with ALL were enrolled. 3 g or 5 g MTX/m^2^ was administered over 24 hours. Serum MTX concentrations were determined in 24, 48, and 96 hours after MTX infusion. Serum creatinine concentrations and creatinine clearance rate (CCR) were determined before and 24 and 48 hours after MTX infusion. A total of 173 courses of MTX infusion were administered. The serum creatinine concentrations did not change much after MTX infusion while the CCR was gradually decreased. MTX clearance status was independently related to CCR decrease, with the risk of 8.07 to develop renal impairment in patients with delayed MTX elimination. Serum creatinine concentration, serum creatinine ratio, CCR, and CCR ratio at 24 hours were all related to MTX elimination delay. Patients with serum creatinine level >35.0 *μ*mol/L, creatinine ratio >1.129, or CCR <100.0 mL/min were more likely to undergo MTX elimination delay. In conclusion, MTX could induce transient renal impairment and compromised renal function will delay MTX clearance. The serum creatinine concentration and the ratio and CCR are useful tools for evaluating MTX elimination status.

## 1. Introduction

Methotrexate (MTX) is one of the essential agents for the extramedullary leukemia prophylaxis in acute lymphoblastic leukemia (ALL), non-Hodgkin's lymphoma, and osteosarcoma protocols, which has led to significant improvements in the long-term survival of these patients [1–5]. Genetic polymorphisms relating to MTX metabolism significantly affect long-term survival of patients with ALL [6, 7]. Different from other antineoplastic drugs, the cytotoxic effects of MTX can be partially antagonized by folic acid (leucovorin), which allows us to use the high-dose intravenous MTX up to 33.6 g/m^2^ [3, 8]. However, MTX related toxicity remains an important issue even by now, which includes nephrotoxicity, hepatotoxicity, gastrointestinal mucositis, bone marrow suppression, and neurotoxicity [9–11]. The toxicity is even severer in patients with elimination delay of MTX [12].

In order to predict severe adverse events during high-dose MTX (HD-MTX) chemotherapy, many pharmacokinetic models of HD-MTX infusion have been developed [13–15]; however, these models are complicated and not very helpful in clinical practice. On the one hand, besides MTX itself, the patient's pharmacogenomics, organ function, and the coadministrated anticancer agents may influence the normal elimination of MTX. On the other hand, these models were not informative enough for patients with delayed MTX elimination. Therefore, serum MTX concentration monitoring is still a standard approach for identifying patients at high risk of developing toxicity. However, there are still many oncological institutions that cannot carry out serum MTX concentration measurement in China, so it is of great importance to find a simple and convenient way to screen out patients at high risk of developing toxicity.

It is well known that renal clearance is the principal pathway of MTX elimination, and its elimination appears to be related to renal function [16, 17]. On the other hand, nephrotoxicity is one of the most frequently reported side effects of HD-MTX infusion, especially in patients with delayed MTX elimination. Thus, it is important to illustrate the association between renal function and MTX elimination, which could guide the clinical decision making to prevent severe adverse events.

The purpose of this study was to evaluate the change of renal function after administration of HD-MTX by parameters such as serum creatinine and creatinine clearance rate (CCR) and to find out helpful parameters that can be used to predict delayed MTX elimination and to prevent renal impairment.

## 2. Materials and Methods

### 2.1. Patients

This study was approved by the Medical Ethics Committee of the Children's Hospital of Zhejiang University School of Medicine. Fifty-four patients with newly diagnosed ALL from April 2014 to February 2015 were enrolled in this study after informed consent was obtained. All of these patients were treated with modified National Protocol of Childhood Leukemia in China 2006 (NPCLC-ALL2006) [18]. Of the 54 patients, 33 were boys and 21 were girls, with a median age of 5.4 years (range from 2.2 years to 15.0 years). According to risk stratification, 12 patients were from high risk group, 9 from intermediate risk group, and 33 from low risk group. The diagnostic and the risk grouping criteria were as previously described [18]. A total of 173 courses of MTX were administered.

### 2.2. MTX Administration and Serum MTX Concentration Determination

The total MTX dose was 3 g/m^2^ for low risk group and 5 g/m^2^ for intermediate and high risk groups. For each course, 1/6 of the total MTX dose (maximum to 0.5 g) was given intravenously in the first hour and the rest was administered evenly during the subsequent 23 hours. Intravenous hydration and urinary alkalinization were performed one day before HD-MTX administration at doses of 2000–3000 mL/m^2^/d for low risk patients and 3000–4000 mL/m^2^/d for intermediate and high risk patients with 5% of sodium bicarbonate at a dosage of 5 mL/kg/d. Intravenous hydration and urinary alkalinization were continued during and after MTX infusion until the MTX serum concentration was <0.1 *μ*mol/L.

The serum MTX concentration was determined by fluorescent polarization immunoassay at 24, 48, and 96 hours after MTX administration and was further detected until the concentration was below 0.1 *μ*mol/L. Patients with serum MTX concentrations ≥1.0 *μ*mol/L at 48 hours or ≥0.1 *μ*mol/L at 96 hours were considered as elimination delay [12]. Leucovorin was administered on the time of 42 hours after MTX administration as an intravenous bolus every 6 hours for 3 to 8 doses or until the serum MTX concentration was <0.1 *μ*mol/L.

### 2.3. Assessment of Renal Function

Creatinine clearance using 24-hour urine collection had been measured in all patients before and 24 and 48 hours after the initiation of HD-MTX treatment. A portion of the collection was used to measure the urinary creatinine concentration, and a venous blood sample was drawn for the measurement of serum creatinine level in the last hour of urine sample collection. The following formula was used to calculate the CCR: CCR = [urine creatinine (mg/dL) × 24 hour urine volume (mL) × 1.73 m^2^]/[body surface area (m^2^) × serum creatinine (mg/dL) × 1440]. Serum creatinine ratio was calculated using the following formula: serum creatinine ratio = serum creatinine (mg/dL) at 48 hours or 96 hours/serum creatinine (mg/dL) before MTX infusion and CCR ratio = CCR at 48 hours or 96 hours/CCR before MTX infusion.

### 2.4. Statistical Analysis

The comparisons of patients' demographic features and creatinine metabolism parameters between normal and delayed MTX elimination groups were performed by Mann-Whitney *U* test. Increase of serum creatinine level and decrease of CCR in every group were compared by Wilcoxon signed rank test. The selection of parameters related to renal impairment (CCR decrease >50%) was performed using binary logistic regression model. The correlations between creatinine metabolism parameters and serum MTX concentrations were determined by Spearman's rank correlation analysis. The predictive power of creatinine level, ratio, and CCR for MTX elimination were evaluated by receiver's operating characteristic (ROC) curve; the odds ratio (OR) and its 95% confidence interval (95% CI) were calculated by *χ* ^2^ test. All analyses were performed on SPSS12.0 software. A *P* value < 0.05 (two-tailed) was considered to be of statistical significance.

## 3. Results

### 3.1. Patients' Characteristics

A total of 173 courses of MTX infusion were observed in this study. As for dosages, 113 courses were 3 g/m^2^ and 60 courses were 5 g/m^2^. Forty-eight episodes of elimination delay were observed, accounting for 27.7% of total courses. The rate of elimination delay showed no difference between 3 g/m^2^ group (31.9%, 36/113) and 5 g/m^2^ group (20.0%, 12/60) (*P* = 0.097). Gastrointestinal adverse reaction such as nausea and vomiting, oral mucositis, and bone marrow depression were the most common toxic effects. Thirty-two episodes of febrile neutropenia occurred. Hepatic impairment was found in 63 infusions. No fatal adverse event occurred in this cohort. We compared the demographic features and some potential characteristics that may be correlated with MTX elimination, including age, height, weight, body surface area (BSA), serum creatinine level, serum creatinine ratio, urine output (adjusted to BSA), and CCR. As shown in Table 1, patient demographic characteristics, including gender, age, weight, and BSA, were comparable between the delayed elimination group and the normal elimination group as well. However, patients with MTX clearance delay showed higher serum creatinine concentrations, higher serum creatinine ratios, and severer CCR decrease at 24 hours and 48 hours.

### 3.2. Renal Impairment in HD-MTX Chemotherapy

**Figure 1:**
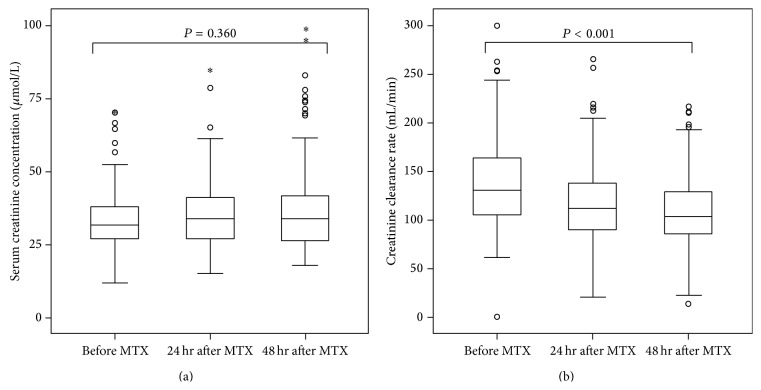
Serum creatinine (a) and creatinine clearance rate (CCR) change (b) after MTX infusion.

We first assessed renal function change after HD-MTX infusion by serum creatinine and CCR. As shown in Figure 1, the serum creatinine concentrations did not change much in 24 hours and 48 hours after HD-MTX infusion when compared with that before chemotherapy (median concentrations, 0 hours versus 24 hours versus 48 hours: 31.7 *μ*mol/L versus 33.5 *μ*mol/L versus 33.8 *μ*mol/L, *P* = 0.360). However, the CCR level was gradually decreased after HD-MTX infusion (median concentrations, 0 hours versus 24 hours versus 48 hours: 131.2 mL/min versus 112.3 mL/min versus 102.7 mL/min, *P* < 0.001), indicating the renal function impairment after HD-MTX chemotherapy. It also revealed that CCR was a more sensitive marker to assess renal function in this situation.

### 3.3. Delayed MTX Elimination Was Significantly Related to Renal Impairment

**Figure 2:**
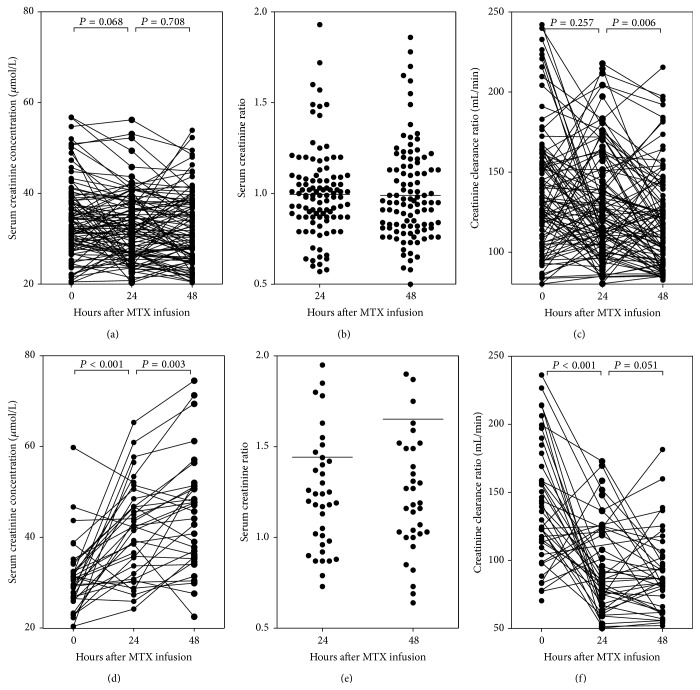
Comparison of renal function in normal and delayed MTX elimination. In normal MTX elimination patients, serum creatinine concentration (a) and creatinine ratio (b) were comparable while the CCR was slightly decreased 24 hours after MTX infusion (c). In delayed MTX elimination patients, serum creatinine concentration (d) and creatinine ratio (e) were gradually increased while CCR was decreased (f).

As CCR was a good marker for renal function, we then defined CCR decrease more than 50% in 48 hours compared with that before HD-MTX infusion as renal impairment. Parameters probably related to renal impairment were collected in Table 2, including age, body surface area, MTX dose, creatinine level before MTX infusion, MTX concentration in 24 hours, and MTX clearance status in 48 hours. Only MTX clearance status was independently related to CCR decrease, with the risk of 8.07 (95% CI, 2.57–25.32) to develop renal impairment in patients with delayed MTX elimination. We further investigated the serum creatinine, serum creatinine ratio, and CCR change during HD-MTX infusion in normal MTX elimination group and delayed MTX elimination group, respectively (Figure 2), and found that both serum creatinine and serum creatinine ratio were significantly increased while CCR was markedly decreased in delayed MTX elimination group. However, only CCR was slightly decreased between 24 hours and 48 hours after MTX infusion in normal MTX elimination group.

### 3.4. Biomarkers to Predict Delayed MTX Elimination

**Figure 3:**
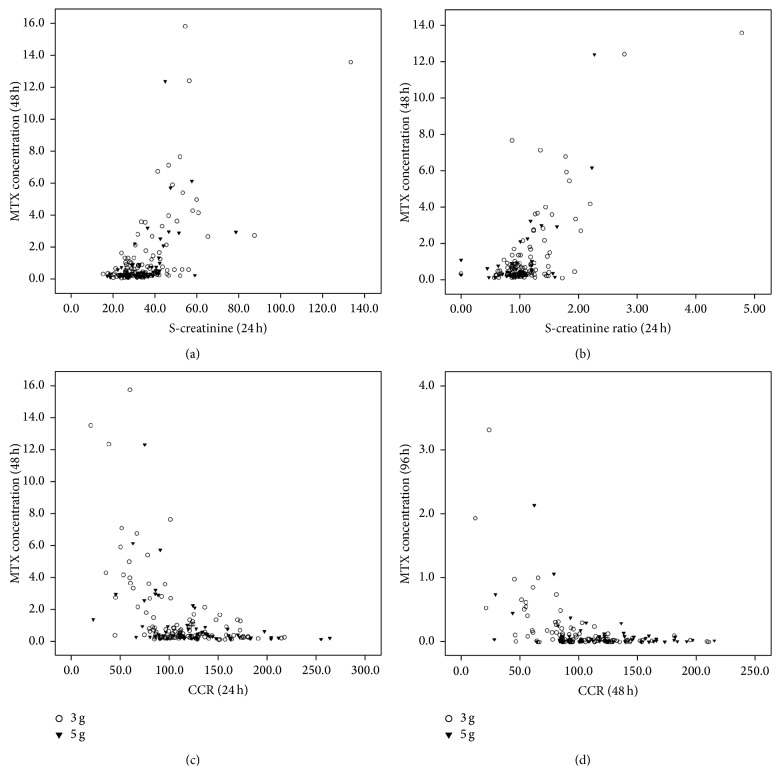
The correlations between serum creatinine concentration (*μ*mol/L), the ratio of serum creatinine level in 24 hours to creatinine level before MTX infusion (S-creatinine ratio), creatinine clearance rate (CCR, mL/min), and serum MTX concentration (*μ*mol/L) in 3 g/m^2^ (unfilled circle) and 5 g/m^2^ groups (filled inverted triangle).

As MTX elimination delay was significantly related to renal impairment, if the physician could predict MTX elimination delay in an early time and give proper intervention, the renal toxicity and other complications related to MTX elimination delay might be alleviated. In order to screen out parameters that can be used to predict MTX clearance delay, the correlations between the serum metabolism and MTX concentrations at 48 hours and 96 hours were analyzed. As shown in Figure 3, serum creatinine concentration at 24 hours was positively related to the MTX concentrations at 48 hours (*r* = 0.525, *P* < 0.001) and 96 hours (*r* = 0.577, *P* < 0.001). Moreover, serum creatinine ratio at 24 hours seemed more closely related to the MTX concentrations at 48 hours (*r* = 0.749, *P* < 0.001) and 96 hours (*r* = 0.764, *P* < 0.001), respectively. CCR at 24 hours was inversely related to the MTX concentration at 48 hours (*r* = −0.398, *P* < 0.001) and 96 hours (*r* = −0.331, *P* < 0.001), and CCR ratio at 24 hours was only slightly related to the MTX concentration at 48 hours (*r* = −0.356, *P* < 0.001) and 96 hours (*r* = −0.289, *P* < 0.001).

As all the four parameters were related to MTX elimination delay, we then further assessed their function in predicting MTX elimination delay. As shown in Table 3, serum creatinine 24 hours after MTX infusion, serum creatinine ratio (24 hours), CCR (24 hours), and CCR ratio (24 hours) all predict MTX elimination delay by ROC analysis, with the accuracy around 75%. We found that patients with serum creatinine level >35.0 *μ*mol/L, serum creatinine ratio >1.129, CCR level <100.0 mL/min, and CCR ratio <0.824 more likely experienced MTX elimination delay. Serum creatinine ratio seemed to be the best marker for the prediction. When serum creatinine concentration was >46.0 *μ*mol/L, serum creatinine ratio was >1.5, CCR level was <80.0 mL/min, or/and CCR ratio was <0.59, the probability for elimination delay was more than 95%.

## 4. Discussion

The present study shows that HD-MTX administration induces temporal CCR decrease, and the elevated creatinine level, the high creatinine ratio, and the decrease of CCR are closely related to delayed MTX elimination. These findings indicate that HD-MTX can induce transient kidney impairment and compromised kidney function will delay MTX clearance. However, none of the parameters for renal function measured before the start of HD-MTX infusion was found to be correlated with MTX elimination.

Renal clearance is the main pathway for MTX elimination. About 70%~90% of the dose is excreted unchanged in the urine [19]. It has been reported that increased serum creatinine level during HD-MTX infusion is often associated with delayed MTX elimination [16], which is consistent with the present study. Nevertheless, this study also found that patients experienced a short time decrease of CCR after MTX infusion, which confirmed that HD-MTX administration can induce transient renal function impairment [20, 21]. However, CCR is a more sensitive parameter for predicting kidney impairment when compared with serum creatinine level, especially for patients with normal MTX elimination. Of the “normal” patients, although minimal impairment of renal function occurred according to CCR, the creatinine showed no elevation, which was similar to Skärby et al.'s and Ylinen et al.'s results [16, 22]. This may be attributed to the compensation of kidney to maintain serum creatinine concentrations stable.

The relationship between serum MTX concentration and creatinine clearance was controversial. Studies from Evans, Relling, and Joannon all reported that there was no correlation between serum MTX concentration and creatinine clearance [2, 11, 23]. In these studies, the MTX dosages were 1 g, 0.9~3.7 g, and 1~2 g, respectively, which were lower than the dosage in the present study. The MTX concentrations in 24 and 48 hours were much lower as well, which implied that patients in these studies might undergo a lesser degree of renal function impairment according to Hempel et al. [21]. As kidney has powerful compensatory capacity [24], slight impairment has minimal influence on its clearance of MTX. Thus the dose-responses effect of CCR variation on MTX elimination may not be obvious to a certain extent in these patients. This was similar to normal elimination group in this study.

This study illustrated the relationship between renal function and MTX elimination. According to the creatinine change and CCR level, physicians could evaluate the MTX elimination status 24 hours before MTX concentration report and adjust alkalization and hydration as early as possible, so that the toxicity of MTX could be reduced. In some institutions where MTX concentration detection cannot be performed, creatinine and CCR investigation can be a useful tool to find out patients with high risk of delayed MTX elimination. For patients with normal renal function, regular alkalization, hydration, and leucovorin can be used. However, for patients with high creatinine level/ratio or low CCR level, they are likely to undergo delayed MTX elimination, so the remedies to improve renal function and increase MTX clearance should be applied accordingly.

## Figures and Tables

**Table 1:** Comparison of patients' characteristics and serum MTX concentrations between patients with and without MTX elimination delay.

Characteristics	Whole cohort	Number ratio	Normal elimination	Delayed elimination	*P* value
Height (cm)	110	123 versus 50	110.0	108.5	0.629
Weight (kg)	19.0	123 versus 50	20.0	19.0	0.493
Age (year)	5.2	123 versus 50	5.4	5.0	0.461
BSA (m^2^)	0.85	123 versus 50	0.75	0.75	0.615
S-creatinine 0 h (*μ*mol/L)	31.7	110 versus 41	32.8	30.9	0.083
S-creatinine 24 h (*μ*mol/L)	33.5	120 versus 48	31.6	42.5	**<0.001**
S-creatinine 48 h (*μ*mol/L)	33.8	122 versus 50	30.5	47.4	**<0.001**
S-creatinine ratio 24 h	1.02	110 versus 41	0.98	1.26	**<0.001**
S-creatinine ratio 48 h	1.01	110 versus 41	0.95	1.39	**<0.001**
CCR_0 h (mL/min)	131.3	111 versus 41	130.1	139.5	0.424
CCR_24 h (mL/min)	111.3	118 versus 48	120.2	85.1	**<0.001**
CCR_48 h (mL/min)	102.7	120 versus 49	114.1	81.7	**<0.001**
CCR_96 h (mL/min)	97.7	12 versus 17	113.8	86.4	**0.008**
*C*_MTX__24 h (*μ*mol/L)	59.8	123 versus 50	54.5	70.5	**<0.001**
*C*_MTX__48 h (*μ*mol/L)	0.43	123 versus 50	0.32	2.81	**<0.001**
*C*_MTX__96 h (*μ*mol/L)	0.04	115 versus 49	0.02	0.25	**<0.001**

S-creatinine 0 h: serum creatinine level before MTX infusion; S-creatinine 24 h: serum creatinine level in 24 hours after MTX infusion; S-creatinine 48 h: serum creatinine level in 48 hours after MTX infusion; S-creatinine ratio 24 h: the ratio of serum creatinine level in 24 hours to creatinine level before MTX infusion; S-creatinine ratio 48 h: the ratio of serum creatinine level in 48 hours to creatinine level before MTX infusion; CCR_0 h: creatinine clearance rate before MTX infusion; CCR_24 h: creatinine clearance rate in 24 hours after MTX infusion; CCR_48 h: creatinine clearance rate in 48 hours after MTX infusion; CCR_96 h: creatinine clearance rate in 96 hours after MTX infusion; *C*_MTX__24 h: serum MTX concentration in 24 hours after MTX infusion; *C*_MTX__48 h: serum MTX concentration in 48 hours after MTX infusion; *C*_MTX__96 h: serum MTX concentration in 96 hours after MTX infusion.

**Table 2:** Possible parameters related to CCR decrease > 50%.

Parameters	Standard	Number ratio	Relative risk	95% CI	*P* value
Age ≥10 years	<10 years	41 versus 132	0.81	0.09–7.30	0.852
Body surface area ≥1.0 m^2^	<1.0 m^2^	51 versus 122	1.77	0.24–12.96	0.573
MTX dosage (≥5 g/m^2^)	<5 g/m^2^	60 versus 113	0.98	0.24–4.10	0.979
S-creatinine 0 h ≥35 *μ*mol/L	<35 *μ*mol/L	58 versus 115	0.31	0.07–1.44	0.134
*C*_MTX__24 h ≥65 *μ*mol/L	<65 *μ*mol/L	74 versus 99	0.83	0.26–2.65	0.758
Delayed MTX elimination	Normal elimination	53 versus 120	8.07	2.57–25.32	**<0.001**

S-creatinine 0 h: serum creatinine level before MTX infusion; *C*_MTX__24 h: serum MTX concentration in 24 hours after MTX infusion.

**Table 3:** The predictive value of S-creatinine, S-creatinine ratio, and CCR for delayed MTX elimination.

Parameters	AUC	*P* value	Cut-off	Sensitivity	Specificity
S-creatinine 24 h	0.756 (0.672–0.840)	<0.001	>35.0	68.6%	70.1%
S-creatinine ratio 24 h	0.759 (0.668–0.850)	<0.001	>1.129	69.8%	80.0%
CCR_24 h	0.771 (0.688–0.854)	<0.001	<100.0	68.6%	74.1%
CCR ratio 24 h	0.728 (0.626–0.829)	<0.001	<0.824	67.4%	73.1%
S-creatinine 0 h	0.422 (0.324–0.519)	0.130			
CCR_0 h	0.509 (0.409–0.608)	0.859			

S-creatinine 24 h: serum creatinine level in 24 hours after MTX infusion; S-creatinine ratio 24 h: the ratio of serum creatinine level in 24 hours to creatinine level before MTX infusion; CCR_24 h: creatinine clearance rate in 24 hours after MTX infusion; CCR ratio 24 h: the ratio of CCR in 24 hours to CCR before MTX infusion; S-creatinine 0 h: serum creatinine level before MTX infusion; CCR_0 h: creatinine clearance rate before MTX infusion.

## Abbreviations

MTX: Methotrexate
ALL: Acute lymphoblastic leukemia
CCR: Creatinine clearance rate.
